# Supplementary material for: 30-Minute Highly Multiplexed VaxArray Immunoassay for Pneumococcal Vaccine Antigen Characterization
Source: Vaccines (Basel). 2022 Nov 19;10(11):1964. doi: 10.3390/vaccines10111964 (PMC9693921; doi:10.3390/vaccines10111964)
Supplement: Supplementary file 1 [file vaccines-10-01964-s001.zip › 2022-11-18-Pneumo Manuscript_Supplemental FIGURES and tables.pdf]

Supplemental Table S1: Reactivity of conjugates using 23-Mix Primary Detection Label. Signal to blank ratios (S/Bl) generated on the VaxArray 23-valent Pneumococcal Assay for monovalent Pfizer- and EuBiologics (EuB)-provided conjugates at 2 µg/mL using the 23-Mix Primary Detection Label. Columns represent each serotype-specific capture antibody, and rows represent each conjugate tested. Text in bold green shows S/Bl>3 generated for the matched conjugate and capture antibody. Text in bold red shows S/Bl<3 generated for the matched conjugate and capture antibody indicating low reactivity. Text in bold black shows S/Bl>3 generated on off-target capture antibody indicating cross-reactivity.

		**Serotype-Specific Microarry Capture Antibody**																						
		**S1**	**S2**	**S3**	**S4**	**S5**	**S6A**	**S6B**	**S7F**	**S8**	**S9N**	**S9V**	**S10A**	**S12F**	**S14**	**S15B**	**S17F**	**S18C**	**S19A**	**S19F**	**S20**	**S22F**	**S23F**	**S33F**
Pfizer	S1	**5.8**	1.0	1.0	1.0	0.9	1.0	0.9	0.9	0.9	1.0	1.0	0.9	1.0	1.0	1.0	1.0	0.9	0.9	1.1	1.1	1.1	1.1	1.2
EuB	S1	**12.6**	1.1	0.9	1.0	0.9	0.9	0.7	0.6	0.5	0.8	0.9	0.8	0.6	0.6	0.7	0.7	0.7	0.6	0.8	1.0	1.1	0.9	1.0
Pfizer	S3	0.9	1.1	**6.8**	1.0	0.9	1.0	0.9	1.0	0.9	0.9	0.9	0.9	1.0	1.0	1.1	1.0	1.0	1.0	1.0	1.1	1.1	1.0	1.3
EuB	S3	1.1	1.1	**7.7**	1.2	1.1	1.0	1.1	1.1	1.1	1.0	1.0	1.1	1.1	1.2	1.2	1.3	1.2	1.1	1.0	1.1	1.7	1.1	1.3
Pfizer	S4	1.0	1.3	1.0	**9.3**	1.1	1.1	1.0	1.0	1.0	1.0	1.0	1.0	1.1	1.0	1.1	1.0	1.1	1.1	1.1	1.2	1.1	1.1	1.3
EuB	S4	1.2	1.3	1.3	**9.9**	1.2	1.2	1.2	1.4	1.1	1.1	1.1	1.2	1.2	1.1	1.4	1.4	1.3	1.1	1.1	1.3	1.1	1.2	1.4
Pfizer	S5	1.0	1.1	1.0	0.9	**11.8**	1.0	0.9	0.9	1.2	1.0	1.0	1.0	1.0	1.0	1.0	1.0	0.9	0.9	1.0	1.1	1.1	1.1	1.1
EuB	S5	1.2	1.3	1.2	1.3	**12.9**	1.3	1.2	1.2	1.5	1.1	1.1	1.1	1.2	1.2	1.3	1.3	1.3	1.2	1.2	1.1	1.2	1.1	1.3
Pfizer	S6A	1.1	1.0	1.0	0.9	0.9	**8.3**	1.0	0.9	0.9	0.9	1.0	1.1	1.0	1.0	1.0	0.9	0.9	0.9	1.1	1.2	1.1	1.1	1.1
EuB	S6A	1.1	1.2	1.2	1.2	1.1	**7.7**	1.1	1.1	1.0	1.0	1.0	1.1	1.0	1.0	1.2	1.1	1.1	1.0	1.0	1.0	1.0	1.0	1.1
Pfizer	S6B	1.1	1.2	1.1	1.1	1.1	1.2	**8.9**	1.1	1.1	1.1	1.0	1.0	1.0	1.1	1.2	1.1	1.2	1.1	1.0	1.1	1.0	1.0	1.2
EuB	S6B	1.1	1.2	1.1	1.1	1.0	1.2	**8.7**	1.1	1.0	1.1	1.0	1.0	1.0	1.0	1.1	1.1	1.2	1.0	1.0	1.2	1.0	1.1	1.2
Pfizer	S7F	1.0	1.0	0.9	1.1	1.0	1.1	1.0	**9.9**	1.0	1.0	1.0	1.1	1.0	1.0	1.0	1.0	1.0	1.1	1.0	1.2	1.0	1.0	1.2
EuB	S7F	1.1	1.1	1.2	1.2	1.1	1.2	1.1	**13.6**	1.1	1.0	1.0	1.1	1.0	1.0	1.1	1.1	1.3	1.0	1.0	1.0	1.0	0.9	1.0
Pfizer	S9V	1.0	1.1	0.9	1.0	1.0	1.1	1.0	1.0	1.0	1.0	**14.3**	1.2	1.0	1.0	1.0	1.0	1.0	1.1	1.0	1.0	1.0	0.9	1.2
EuB	S9V	1.3	1.8	1.7	1.9	1.2	1.4	1.3	1.1	1.2	1.3	**10.3**	1.3	1.4	1.2	1.7	1.8	1.6	1.1	1.1	1.9	1.3	2.0	2.0
Pfizer	S14	1.6	1.1	0.9	0.9	1.0	1.1	1.0	1.0	1.0	1.0	1.0	1.1	1.1	**11.8**	1.0	1.0	1.1	1.1	1.0	1.1	1.0	0.8	1.3
EuB	S14	2.0	2.2	1.7	2.2	1.3	1.6	1.5	1.2	1.3	1.4	1.3	1.5	1.4	**14.9**	2.1	2.0	1.9	1.3	1.1	2.1	1.4	2.1	2.1
Pfizer	S18C	1.0	1.0	0.9	1.0	1.0	1.0	1.0	1.0	0.9	1.0	1.0	1.0	1.0	1.0	1.0	1.0	**1.1**	1.0	1.0	1.0	1.0	0.9	1.1
EuB	S18C	1.4	2.0	1.6	1.9	1.3	1.5	1.4	1.1	1.1	1.4	1.2	1.3	1.4	1.2	1.9	2.0	**13.7**	1.2	1.2	1.8	1.3	2.0	2.1
Pfizer	S19A	1.0	0.9	0.8	0.8	1.0	0.9	1.0	1.0	0.9	1.0	1.0	1.0	1.0	1.0	0.9	0.9	1.0	**9.3**	1.0	0.9	1.0	**5.9**	1.0
EuB	S19A	1.4	2.0	1.7	2.0	1.3	1.5	1.4	1.1	1.3	1.4	1.3	1.3	1.4	1.2	1.7	1.8	1.7	**16.1**	1.1	1.8	1.3	1.8	1.8
Pfizer	S19F	1.0	0.9	1.0	1.0	0.9	0.9	1.0	1.0	0.9	1.0	0.9	1.1	1.1	0.9	1.2	1.1	1.1	1.0	**12.7**	1.3	1.0	1.1	1.3
EuB	S19F	1.5	2.2	1.9	2.1	1.3	1.6	1.4	1.2	1.2	1.5	1.3	1.4	1.4	1.2	1.7	1.7	1.9	1.2	**16.1**	1.9	1.4	1.9	1.9
EuB	S22F	0.9	0.9	0.9	0.9	0.9	0.9	0.9	0.9	0.9	0.9	0.9	0.9	0.9	0.9	0.8	0.9	0.9	0.9	0.9	0.9	**10.5**	0.9	0.9
Pfizer	S23F	0.9	0.8	0.9	0.9	0.9	1.0	1.0	0.9	0.9	1.0	0.9	0.9	1.0	0.9	1.1	1.2	1.1	1.0	0.9	1.1	1.0	**5.2**	1.2
EuB	S23F	0.9	1.0	1.0	0.9	0.9	0.9	0.9	0.9	0.9	0.8	0.9	0.9	0.9	1.0	0.9	0.9	0.9	0.9	0.9	0.9	0.9	**3.3**	1.0

Supplemental Table S2: Reactivity of conjugates using anti-CRM197 Primary Detection Label. Signal to blank ratios (S/Bl) generated on the VaxArray 23-valent Pneumococcal Assay for monovalent Pfizer- and EuBiologics (EuB)-provided conjugates at 2 µg/mL labeled with anti-CRM197 Primary Detection Label. Columns represent each serotype-specific capture antibody, and rows represent each conjugate tested. Text in bold green shows S/Bl>3 generated for the matched conjugate and capture antibody. Text in bold black shows S/Bl>3 generated on off-target capture antibody indicating cross-reactivity.

		Serotype-Specific Microarry Capture Antibody																						
		**S1**	**S2**	**S3**	**S4**	**S5**	**S6A**	**S6B**	**S7F**	**S8**	**S9N**	**S9V**	**S10A**	**S12F**	**S14**	**S15B**	**S17F**	**S18C**	**S19A**	**S19F**	**S20**	**S22F**	**S23F**	**S33F**
Pfizer	S1	**12.0**	1.0	1.0	1.0	1.0	1.0	1.0	1.0	1.0	1.0	1.0	1.0	1.0	1.0	1.0	1.0	1.0	1.0	1.0	1.0	1.0	1.0	1.0
EuB	S1	**5.8**	1.0	1.0	1.0	1.0	1.0	1.0	1.0	1.0	1.0	1.0	1.1	1.0	1.1	1.0	1.0	1.0	1.0	1.0	1.0	1.1	1.0	1.0
Pfizer	S3	1.1	1.0	**13.2**	1.0	1.0	1.0	1.0	1.0	1.0	1.1	1.0	1.0	1.0	1.0	1.0	1.0	1.1	1.0	1.0	1.0	1.0	1.0	1.0
EuB	S3	1.1	1.1	**11.9**	1.1	1.1	1.1	1.1	1.1	1.1	1.1	1.1	1.1	1.1	0.9	1.1	1.1	1.1	1.1	1.1	1.0	1.7	1.1	1.0
Pfizer	S4	1.1	1.0	1.1	**18.1**	1.1	1.1	1.0	1.0	1.0	1.1	1.1	1.1	1.1	1.1	1.1	1.1	1.1	1.0	1.1	1.1	1.0	1.1	1.1
EuB	S4	1.1	1.0	1.0	**12.1**	1.1	1.1	1.0	1.1	1.0	1.1	1.0	1.1	1.1	1.2	1.0	1.1	1.0	1.0	1.1	1.0	1.1	1.1	1.0
Pfizer	S5	1.0	1.0	1.1	1.1	**13.4**	1.1	1.0	1.0	1.1	1.1	1.0	1.0	1.0	0.9	1.0	1.0	1.0	1.0	1.0	1.0	1.0	1.0	1.0
EuB	S5	1.1	1.0	1.0	1.0	**3.6**	1.0	1.0	1.0	1.1	1.0	1.0	1.0	1.1	1.1	1.0	1.0	1.0	1.0	1.0	1.0	1.0	1.0	1.0
Pfizer	S6A	1.0	1.0	1.0	1.0	1.0	**12.4**	1.0	1.0	1.0	1.1	1.0	1.0	1.0	1.0	1.0	1.0	1.0	1.0	1.1	1.0	1.0	1.0	1.0
EuB	S6A	1.0	1.0	1.0	1.0	1.1	**11.5**	1.1	1.0	1.0	1.0	1.1	1.0	1.0	1.1	1.0	1.0	1.0	1.0	1.1	1.0	1.0	1.0	1.0
Pfizer	S6B	1.0	1.0	1.0	1.0	1.0	0.9	**12.5**	0.9	1.0	1.0	1.0	1.0	1.0	1.1	0.9	1.0	1.0	1.0	1.0	1.0	1.0	1.0	1.0
EuB	S6B	1.0	1.0	1.0	1.0	1.0	1.1	**6.3**	1.0	1.0	1.0	1.0	1.0	1.0	1.0	1.0	1.0	1.0	1.0	0.9	1.0	1.0	1.0	1.0
Pfizer	S7F	1.0	1.0	1.0	1.0	1.0	1.0	1.0	**10.1**	1.0	1.0	1.0	0.9	1.0	0.8	1.0	1.0	1.0	1.0	1.0	1.0	0.9	1.0	1.0
EuB	S7F	1.0	1.0	1.0	1.0	1.0	1.0	1.0	**8.7**	1.0	1.0	1.0	1.0	1.0	1.0	1.0	1.0	1.1	1.0	1.0	1.0	1.0	1.0	1.0
Pfizer	S9V	0.9	0.9	0.9	1.0	1.0	0.9	0.9	0.9	0.9	0.9	**17.9**	1.0	1.0	1.0	0.9	0.9	1.0	0.9	0.9	1.0	1.0	0.9	1.0
EuB	S9V	1.0	1.0	1.0	1.0	1.0	1.0	1.0	1.0	1.0	0.9	**15.5**	1.0	1.0	1.1	1.0	1.0	1.0	1.0	1.0	1.0	1.0	1.0	1.0
Pfizer	S14	1.0	1.0	1.0	1.0	1.0	1.0	1.0	1.0	1.0	1.0	1.0	1.0	1.0	**7.0**	1.0	1.0	1.0	1.0	0.9	1.0	1.0	0.9	0.9
EuB	S14	1.1	1.0	0.9	1.0	1.0	1.0	1.0	1.0	1.0	0.9	1.0	1.0	0.9	**5.7**	1.0	1.0	1.0	0.9	0.9	1.0	1.0	1.0	0.9
Pfizer	S18C	1.0	1.0	1.0	1.0	1.0	1.0	1.0	1.0	1.0	0.9	1.0	1.0	1.0	0.9	1.1	1.0	**13.6**	1.0	1.0	1.0	0.9	1.0	1.0
EuB	S18C	1.0	1.0	1.0	1.0	1.1	1.0	1.0	1.1	1.0	1.0	1.0	1.0	1.0	1.1	1.0	1.1	**8.3**	1.0	1.0	1.0	1.0	1.1	1.0
Pfizer	S19A	1.0	0.9	1.0	1.0	1.0	1.0	0.9	0.9	1.0	1.0	1.0	0.9	0.9	1.0	0.9	0.9	0.9	**12.9**	0.9	1.0	1.0	**13.4**	1.0
EuB	S19A	0.9	1.0	0.9	1.0	1.0	1.0	0.9	1.0	1.0	0.9	0.9	0.9	1.0	1.0	1.0	1.0	0.9	**7.0**	1.0	0.9	0.9	1.0	0.9
Pfizer	S19F	1.0	1.0	1.0	1.0	1.0	1.0	1.0	1.0	1.0	1.0	1.0	1.0	1.0	1.0	1.0	1.0	1.0	1.0	**9.9**	0.9	1.0	0.9	1.0
EuB	S19F	1.0	1.0	0.9	0.9	1.0	0.9	1.0	1.0	0.9	1.0	0.9	1.1	1.0	1.0	0.9	1.0	1.0	0.9	**5.5**	1.0	1.0	1.0	0.9
EuB	S22F	1.0	1.0	1.0	0.9	0.9	0.9	0.9	0.9	0.9	0.9	0.9	1.0	0.9	0.8	0.9	0.9	0.9	0.9	0.9	0.9	**6.0**	0.9	0.9
Pfizer	S23F	1.0	1.0	1.0	1.0	1.0	1.0	1.0	1.0	1.0	1.0	1.0	0.9	1.0	0.9	1.0	1.0	1.0	1.0	0.9	1.0	1.0	**16.0**	1.1
EuB	S23F	0.9	0.9	0.9	0.9	0.9	0.9	1.0	0.9	0.9	0.9	0.9	0.9	0.9	0.9	0.9	0.9	0.9	0.9	0.9	0.9	0.9	**8.8**	0.9

Supplemental Table S3: Analytical sensitivity and working range for 13-valent Pfizer conjugates and 14-valent EuBiologics (EuB) conjugates with both 23-Mix and anti-CRM197 Primary Detection Labels. "NA": not applicable due to low signal on S18C capture.

Serotype	Supplier	**Approx. LLOQ** (ng/mL)		**Approx ULOQ** (µg/mL)		**Working Range** (x-fold)	
		**23-Mix Label**	**anti-CRM197 Label**	**23-Mix Label**	**anti-CRM197 Label**	**23-Mix Label**	**anti-CRM197 Label**
**1**	Pfizer	2.4	8.6	0.4	0.8	181	95
	EuB	3.7	13.5	0.8	0.8	226	59
**3**	Pfizer	4.3	6.7	0.6	0.7	144	97
	EuB	28.4	14.8	1.8	1.6	64	109
**4**	Pfizer	2.5	4.2	0.5	0.5	204	123
	EuB	8.5	17.7	0.8	1.1	100	62
**5**	Pfizer	8.6	7.8	0.8	0.8	94	106
	EuB	43.0	98.9	1.7	1.7	39	17
**6A**	Pfizer	47.3	27.4	2.4	1.9	51	71
	EuB	45.5	11.7	2.0	1.4	45	118
**6B**	Pfizer	76.3	26.6	6.6	3.5	86	130
	EuB	78.0	12.8	2.5	1.9	31	148
**7F**	Pfizer	14.4	38.2	3.0	3.3	207	87
	EuB	20.2	39.7	1.8	2.2	90	55
**9V**	Pfizer	29.8	8.9	1.7	1.2	57	129
	EuB	190.7	14.3	2.5	1.6	13	110
**14**	Pfizer	2.5	43.8	1.0	1.7	385	40
	EuB	8.2	41.4	1.1	2.0	129	49
**18C**	Pfizer	NA	8.9	NA	0.6	NA	69
	EuB	18.0	23.8	2.1	1.5	115	63
**19A**	Pfizer	23.9	7.8	1.6	1.1	66	146
	EuB	49.0	14.5	1.9	1.5	39	104
**19F**	Pfizer	13.6	27.3	1.4	1.1	106	41
	EuB	15.5	30.7	1.1	1.3	72	44
**22F**	EuB	14.1	29.0	1.8	1.3	127	43
**23F**	Pfizer	26.2	6.1	1.5	1.0	56	167
	EuB	128.1	9.1	2.6	1.2	21	127

Supplemental Table S4: Accuracy and precision for EuBiologics 15-valent drug product with and without sodium citrate desorption using 23-Mix Primary Detection Label. Text in red indicates values not within 80-120% recovery and < 20% RSD.

Serotype	Expected Conc. (µg/mL)	No Desorption		Desorbed	
		Accuracy (% Recovery)	Precision (%RSD)	Accuracy (% Recovery)	Precision (%RSD)
**S1**	0.18	116%	8%	118%*	14%*
**S3**	0.16	104%	10%	110%	13%
**S4**	0.18	**79%**	**24%**	100%*	12%*
**S5**	0.32	109%	19%	83%	19%
**S6A**	0.28	94%	8%	97%	13%
**S6B**	0.67	96%	9%	105%	11%
**S7F**	0.20	92%	11%	104%	15%
**S9V**	0.20	115%	12%	102%	19%
**S14**	0.13	94%	17%	88%	7%
**S18C**	0.12	**26%**	6%	84%	11%
**S19A**	0.25	93%	5%	101%	11%
**S19F**	0.13	113%	**27%**	91%	6%
**S22F**	0.24	97%	8%	111%	11%
**S23F**	0.27	111%	**65%**	106%	9%
**Overall**		96±23%	16±16%	98±10%	12±4%

*: Data is from 400ms exposure, Std 1 signal saturated at 700ms.

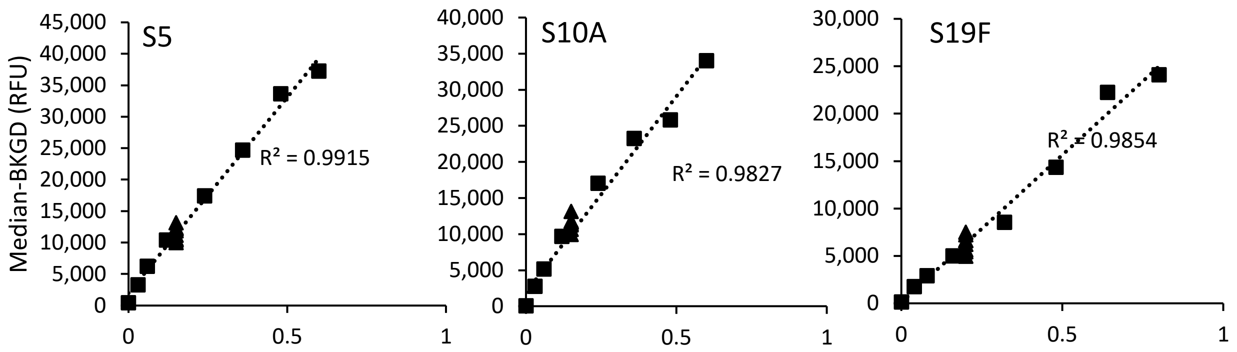

Supplemental Figure S1. Signal response (median signal with background signal subtracted) of samples measured (triangles) and matched standard (squares) for Serotype 5, 10A and 19F in 23-valent Pfizer native sample. Linear fits are dotted lines with the associated correlation efficient indicated.

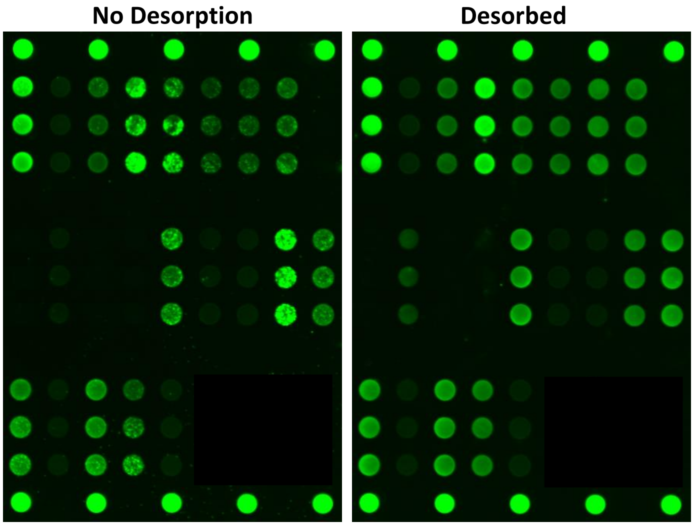

Supplemental Figure S2. Representative fluorescence microarray images of samples under quantification before desorption (left) and post-desorption (right) showing improved microarray spot morphologies.
