# Supplementary material for: 30-Minute Highly Multiplexed VaxArray Immunoassay for Pneumococcal Vaccine Antigen Characterization
Source: Vaccines (Basel). 2022 Nov 19;10(11):1964. doi: 10.3390/vaccines10111964 (PMC9693921; doi:10.3390/vaccines10111964)
Supplement: Supplementary file 1 [file vaccines-10-01964-s001.zip › 2022-11-18-Pneumo Manuscript_Supplemental FIGURES and tables.pptx]

## Slide 1
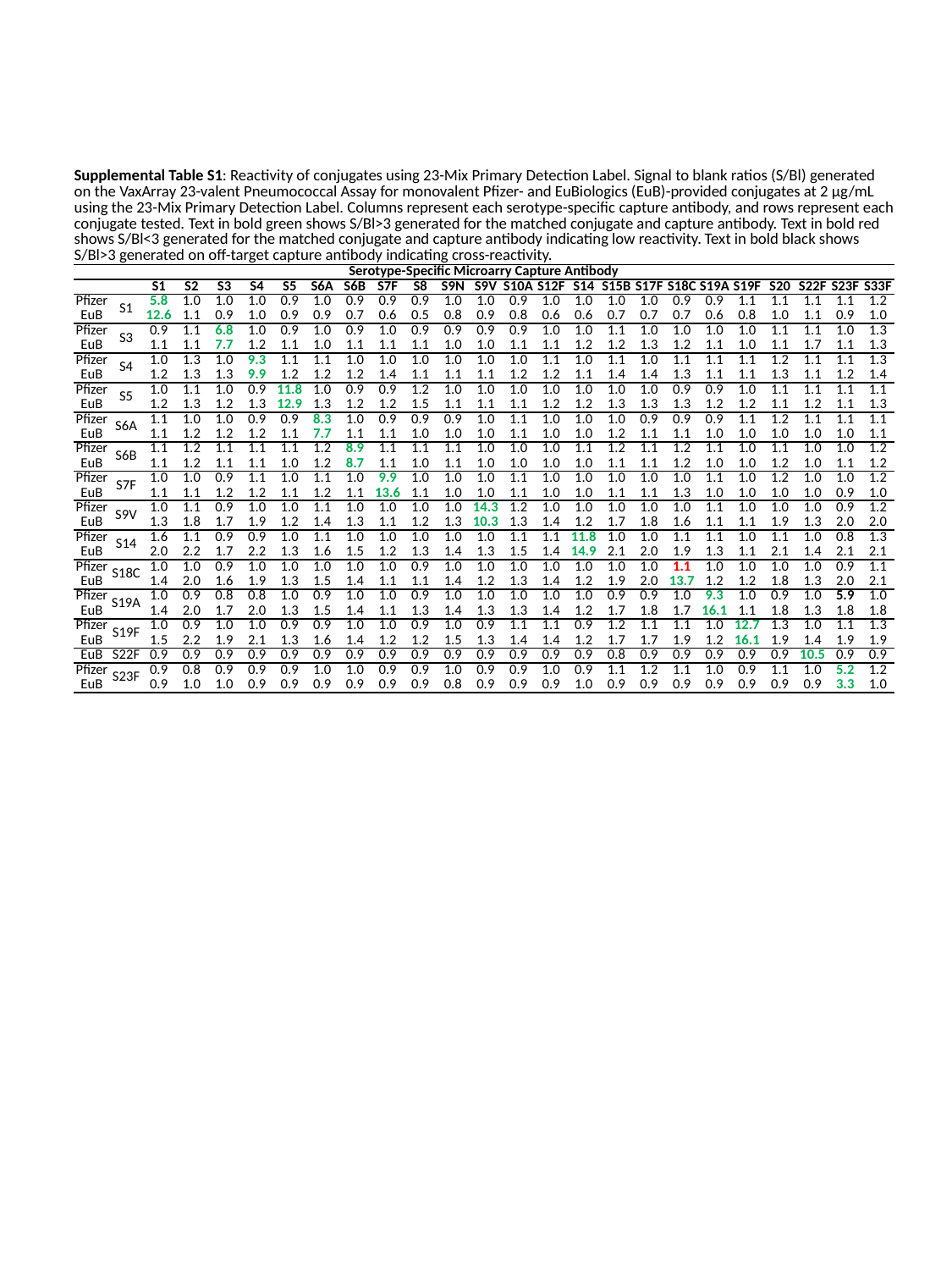

| Supplemental Table S1: Reactivity of conjugates using 23-Mix Primary Detection Label. Signal to blank ratios (S/Bl) generated on the VaxArray 23-valent Pneumococcal Assay for monovalent Pfizer- and EuBiologics (EuB)-provided conjugates at 2 µg/mL using the 23-Mix Primary Detection Label. Columns represent each serotype-specific capture antibody, and rows represent each conjugate tested. Text in bold green shows S/Bl>3 generated for the matched conjugate and capture antibody. Text in bold red shows S/Bl<3 generated for the matched conjugate and capture antibody indicating low reactivity. Text in bold black shows S/Bl>3 generated on off-target capture antibody indicating cross-reactivity. | | | | | | | | | | | | | | | | | | | | | | | | |
| --- | --- | --- | --- | --- | --- | --- | --- | --- | --- | --- | --- | --- | --- | --- | --- | --- | --- | --- | --- | --- | --- | --- | --- | --- |
| Serotype-Specific Microarry Capture Antibody | | | | | | | | | | | | | | | | | | | | | | | | |
| | | S1 | S2 | S3 | S4 | S5 | S6A | S6B | S7F | S8 | S9N | S9V | S10A | S12F | S14 | S15B | S17F | S18C | S19A | S19F | S20 | S22F | S23F | S33F |
| Pfizer | S1 | 5.8 | 1.0 | 1.0 | 1.0 | 0.9 | 1.0 | 0.9 | 0.9 | 0.9 | 1.0 | 1.0 | 0.9 | 1.0 | 1.0 | 1.0 | 1.0 | 0.9 | 0.9 | 1.1 | 1.1 | 1.1 | 1.1 | 1.2 |
| EuB | | 12.6 | 1.1 | 0.9 | 1.0 | 0.9 | 0.9 | 0.7 | 0.6 | 0.5 | 0.8 | 0.9 | 0.8 | 0.6 | 0.6 | 0.7 | 0.7 | 0.7 | 0.6 | 0.8 | 1.0 | 1.1 | 0.9 | 1.0 |
| Pfizer | S3 | 0.9 | 1.1 | 6.8 | 1.0 | 0.9 | 1.0 | 0.9 | 1.0 | 0.9 | 0.9 | 0.9 | 0.9 | 1.0 | 1.0 | 1.1 | 1.0 | 1.0 | 1.0 | 1.0 | 1.1 | 1.1 | 1.0 | 1.3 |
| EuB | | 1.1 | 1.1 | 7.7 | 1.2 | 1.1 | 1.0 | 1.1 | 1.1 | 1.1 | 1.0 | 1.0 | 1.1 | 1.1 | 1.2 | 1.2 | 1.3 | 1.2 | 1.1 | 1.0 | 1.1 | 1.7 | 1.1 | 1.3 |
| Pfizer | S4 | 1.0 | 1.3 | 1.0 | 9.3 | 1.1 | 1.1 | 1.0 | 1.0 | 1.0 | 1.0 | 1.0 | 1.0 | 1.1 | 1.0 | 1.1 | 1.0 | 1.1 | 1.1 | 1.1 | 1.2 | 1.1 | 1.1 | 1.3 |
| EuB | | 1.2 | 1.3 | 1.3 | 9.9 | 1.2 | 1.2 | 1.2 | 1.4 | 1.1 | 1.1 | 1.1 | 1.2 | 1.2 | 1.1 | 1.4 | 1.4 | 1.3 | 1.1 | 1.1 | 1.3 | 1.1 | 1.2 | 1.4 |
| Pfizer | S5 | 1.0 | 1.1 | 1.0 | 0.9 | 11.8 | 1.0 | 0.9 | 0.9 | 1.2 | 1.0 | 1.0 | 1.0 | 1.0 | 1.0 | 1.0 | 1.0 | 0.9 | 0.9 | 1.0 | 1.1 | 1.1 | 1.1 | 1.1 |
| EuB | | 1.2 | 1.3 | 1.2 | 1.3 | 12.9 | 1.3 | 1.2 | 1.2 | 1.5 | 1.1 | 1.1 | 1.1 | 1.2 | 1.2 | 1.3 | 1.3 | 1.3 | 1.2 | 1.2 | 1.1 | 1.2 | 1.1 | 1.3 |
| Pfizer | S6A | 1.1 | 1.0 | 1.0 | 0.9 | 0.9 | 8.3 | 1.0 | 0.9 | 0.9 | 0.9 | 1.0 | 1.1 | 1.0 | 1.0 | 1.0 | 0.9 | 0.9 | 0.9 | 1.1 | 1.2 | 1.1 | 1.1 | 1.1 |
| EuB | | 1.1 | 1.2 | 1.2 | 1.2 | 1.1 | 7.7 | 1.1 | 1.1 | 1.0 | 1.0 | 1.0 | 1.1 | 1.0 | 1.0 | 1.2 | 1.1 | 1.1 | 1.0 | 1.0 | 1.0 | 1.0 | 1.0 | 1.1 |
| Pfizer | S6B | 1.1 | 1.2 | 1.1 | 1.1 | 1.1 | 1.2 | 8.9 | 1.1 | 1.1 | 1.1 | 1.0 | 1.0 | 1.0 | 1.1 | 1.2 | 1.1 | 1.2 | 1.1 | 1.0 | 1.1 | 1.0 | 1.0 | 1.2 |
| EuB | | 1.1 | 1.2 | 1.1 | 1.1 | 1.0 | 1.2 | 8.7 | 1.1 | 1.0 | 1.1 | 1.0 | 1.0 | 1.0 | 1.0 | 1.1 | 1.1 | 1.2 | 1.0 | 1.0 | 1.2 | 1.0 | 1.1 | 1.2 |
| Pfizer | S7F | 1.0 | 1.0 | 0.9 | 1.1 | 1.0 | 1.1 | 1.0 | 9.9 | 1.0 | 1.0 | 1.0 | 1.1 | 1.0 | 1.0 | 1.0 | 1.0 | 1.0 | 1.1 | 1.0 | 1.2 | 1.0 | 1.0 | 1.2 |
| EuB | | 1.1 | 1.1 | 1.2 | 1.2 | 1.1 | 1.2 | 1.1 | 13.6 | 1.1 | 1.0 | 1.0 | 1.1 | 1.0 | 1.0 | 1.1 | 1.1 | 1.3 | 1.0 | 1.0 | 1.0 | 1.0 | 0.9 | 1.0 |
| Pfizer | S9V | 1.0 | 1.1 | 0.9 | 1.0 | 1.0 | 1.1 | 1.0 | 1.0 | 1.0 | 1.0 | 14.3 | 1.2 | 1.0 | 1.0 | 1.0 | 1.0 | 1.0 | 1.1 | 1.0 | 1.0 | 1.0 | 0.9 | 1.2 |
| EuB | | 1.3 | 1.8 | 1.7 | 1.9 | 1.2 | 1.4 | 1.3 | 1.1 | 1.2 | 1.3 | 10.3 | 1.3 | 1.4 | 1.2 | 1.7 | 1.8 | 1.6 | 1.1 | 1.1 | 1.9 | 1.3 | 2.0 | 2.0 |
| Pfizer | S14 | 1.6 | 1.1 | 0.9 | 0.9 | 1.0 | 1.1 | 1.0 | 1.0 | 1.0 | 1.0 | 1.0 | 1.1 | 1.1 | 11.8 | 1.0 | 1.0 | 1.1 | 1.1 | 1.0 | 1.1 | 1.0 | 0.8 | 1.3 |
| EuB | | 2.0 | 2.2 | 1.7 | 2.2 | 1.3 | 1.6 | 1.5 | 1.2 | 1.3 | 1.4 | 1.3 | 1.5 | 1.4 | 14.9 | 2.1 | 2.0 | 1.9 | 1.3 | 1.1 | 2.1 | 1.4 | 2.1 | 2.1 |
| Pfizer | S18C | 1.0 | 1.0 | 0.9 | 1.0 | 1.0 | 1.0 | 1.0 | 1.0 | 0.9 | 1.0 | 1.0 | 1.0 | 1.0 | 1.0 | 1.0 | 1.0 | 1.1 | 1.0 | 1.0 | 1.0 | 1.0 | 0.9 | 1.1 |
| EuB | | 1.4 | 2.0 | 1.6 | 1.9 | 1.3 | 1.5 | 1.4 | 1.1 | 1.1 | 1.4 | 1.2 | 1.3 | 1.4 | 1.2 | 1.9 | 2.0 | 13.7 | 1.2 | 1.2 | 1.8 | 1.3 | 2.0 | 2.1 |
| Pfizer | S19A | 1.0 | 0.9 | 0.8 | 0.8 | 1.0 | 0.9 | 1.0 | 1.0 | 0.9 | 1.0 | 1.0 | 1.0 | 1.0 | 1.0 | 0.9 | 0.9 | 1.0 | 9.3 | 1.0 | 0.9 | 1.0 | 5.9 | 1.0 |
| EuB | | 1.4 | 2.0 | 1.7 | 2.0 | 1.3 | 1.5 | 1.4 | 1.1 | 1.3 | 1.4 | 1.3 | 1.3 | 1.4 | 1.2 | 1.7 | 1.8 | 1.7 | 16.1 | 1.1 | 1.8 | 1.3 | 1.8 | 1.8 |
| Pfizer | S19F | 1.0 | 0.9 | 1.0 | 1.0 | 0.9 | 0.9 | 1.0 | 1.0 | 0.9 | 1.0 | 0.9 | 1.1 | 1.1 | 0.9 | 1.2 | 1.1 | 1.1 | 1.0 | 12.7 | 1.3 | 1.0 | 1.1 | 1.3 |
| EuB | | 1.5 | 2.2 | 1.9 | 2.1 | 1.3 | 1.6 | 1.4 | 1.2 | 1.2 | 1.5 | 1.3 | 1.4 | 1.4 | 1.2 | 1.7 | 1.7 | 1.9 | 1.2 | 16.1 | 1.9 | 1.4 | 1.9 | 1.9 |
| EuB | S22F | 0.9 | 0.9 | 0.9 | 0.9 | 0.9 | 0.9 | 0.9 | 0.9 | 0.9 | 0.9 | 0.9 | 0.9 | 0.9 | 0.9 | 0.8 | 0.9 | 0.9 | 0.9 | 0.9 | 0.9 | 10.5 | 0.9 | 0.9 |
| Pfizer | S23F | 0.9 | 0.8 | 0.9 | 0.9 | 0.9 | 1.0 | 1.0 | 0.9 | 0.9 | 1.0 | 0.9 | 0.9 | 1.0 | 0.9 | 1.1 | 1.2 | 1.1 | 1.0 | 0.9 | 1.1 | 1.0 | 5.2 | 1.2 |
| EuB | | 0.9 | 1.0 | 1.0 | 0.9 | 0.9 | 0.9 | 0.9 | 0.9 | 0.9 | 0.8 | 0.9 | 0.9 | 0.9 | 1.0 | 0.9 | 0.9 | 0.9 | 0.9 | 0.9 | 0.9 | 0.9 | 3.3 | 1.0 |

## Slide 2
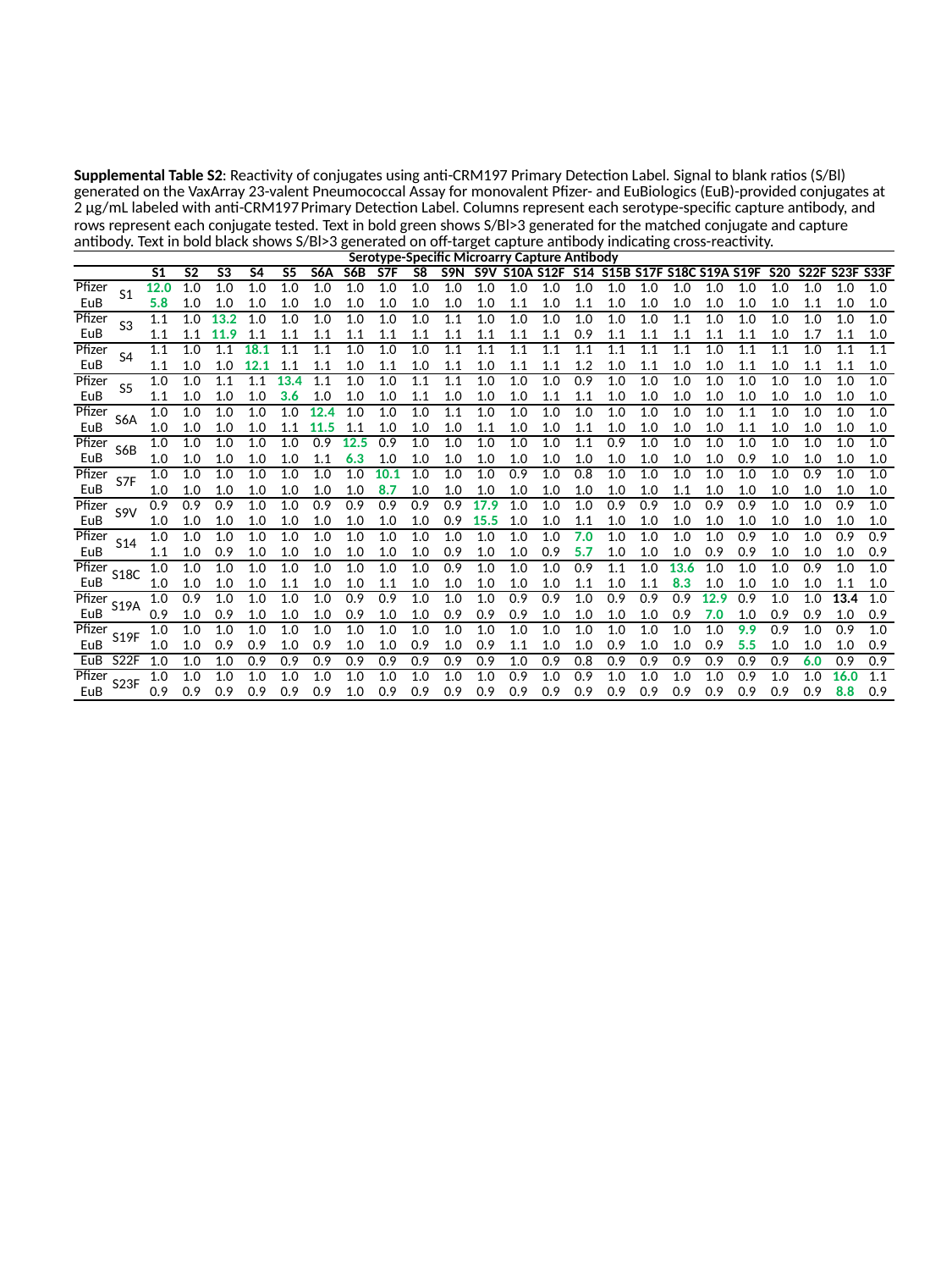

| Supplemental Table S2: Reactivity of conjugates using anti-CRM197 Primary Detection Label. Signal to blank ratios (S/Bl) generated on the VaxArray 23-valent Pneumococcal Assay for monovalent Pfizer- and EuBiologics (EuB)-provided conjugates at 2 µg/mL labeled with anti-CRM197 Primary Detection Label. Columns represent each serotype-specific capture antibody, and rows represent each conjugate tested. Text in bold green shows S/Bl>3 generated for the matched conjugate and capture antibody. Text in bold black shows S/Bl>3 generated on off-target capture antibody indicating cross-reactivity. | | | | | | | | | | | | | | | | | | | | | | | | |
| --- | --- | --- | --- | --- | --- | --- | --- | --- | --- | --- | --- | --- | --- | --- | --- | --- | --- | --- | --- | --- | --- | --- | --- | --- |
| Serotype-Specific Microarry Capture Antibody | | | | | | | | | | | | | | | | | | | | | | | | |
| | | S1 | S2 | S3 | S4 | S5 | S6A | S6B | S7F | S8 | S9N | S9V | S10A | S12F | S14 | S15B | S17F | S18C | S19A | S19F | S20 | S22F | S23F | S33F |
| Pfizer | S1 | 12.0 | 1.0 | 1.0 | 1.0 | 1.0 | 1.0 | 1.0 | 1.0 | 1.0 | 1.0 | 1.0 | 1.0 | 1.0 | 1.0 | 1.0 | 1.0 | 1.0 | 1.0 | 1.0 | 1.0 | 1.0 | 1.0 | 1.0 |
| EuB | | 5.8 | 1.0 | 1.0 | 1.0 | 1.0 | 1.0 | 1.0 | 1.0 | 1.0 | 1.0 | 1.0 | 1.1 | 1.0 | 1.1 | 1.0 | 1.0 | 1.0 | 1.0 | 1.0 | 1.0 | 1.1 | 1.0 | 1.0 |
| Pfizer | S3 | 1.1 | 1.0 | 13.2 | 1.0 | 1.0 | 1.0 | 1.0 | 1.0 | 1.0 | 1.1 | 1.0 | 1.0 | 1.0 | 1.0 | 1.0 | 1.0 | 1.1 | 1.0 | 1.0 | 1.0 | 1.0 | 1.0 | 1.0 |
| EuB | | 1.1 | 1.1 | 11.9 | 1.1 | 1.1 | 1.1 | 1.1 | 1.1 | 1.1 | 1.1 | 1.1 | 1.1 | 1.1 | 0.9 | 1.1 | 1.1 | 1.1 | 1.1 | 1.1 | 1.0 | 1.7 | 1.1 | 1.0 |
| Pfizer | S4 | 1.1 | 1.0 | 1.1 | 18.1 | 1.1 | 1.1 | 1.0 | 1.0 | 1.0 | 1.1 | 1.1 | 1.1 | 1.1 | 1.1 | 1.1 | 1.1 | 1.1 | 1.0 | 1.1 | 1.1 | 1.0 | 1.1 | 1.1 |
| EuB | | 1.1 | 1.0 | 1.0 | 12.1 | 1.1 | 1.1 | 1.0 | 1.1 | 1.0 | 1.1 | 1.0 | 1.1 | 1.1 | 1.2 | 1.0 | 1.1 | 1.0 | 1.0 | 1.1 | 1.0 | 1.1 | 1.1 | 1.0 |
| Pfizer | S5 | 1.0 | 1.0 | 1.1 | 1.1 | 13.4 | 1.1 | 1.0 | 1.0 | 1.1 | 1.1 | 1.0 | 1.0 | 1.0 | 0.9 | 1.0 | 1.0 | 1.0 | 1.0 | 1.0 | 1.0 | 1.0 | 1.0 | 1.0 |
| EuB | | 1.1 | 1.0 | 1.0 | 1.0 | 3.6 | 1.0 | 1.0 | 1.0 | 1.1 | 1.0 | 1.0 | 1.0 | 1.1 | 1.1 | 1.0 | 1.0 | 1.0 | 1.0 | 1.0 | 1.0 | 1.0 | 1.0 | 1.0 |
| Pfizer | S6A | 1.0 | 1.0 | 1.0 | 1.0 | 1.0 | 12.4 | 1.0 | 1.0 | 1.0 | 1.1 | 1.0 | 1.0 | 1.0 | 1.0 | 1.0 | 1.0 | 1.0 | 1.0 | 1.1 | 1.0 | 1.0 | 1.0 | 1.0 |
| EuB | | 1.0 | 1.0 | 1.0 | 1.0 | 1.1 | 11.5 | 1.1 | 1.0 | 1.0 | 1.0 | 1.1 | 1.0 | 1.0 | 1.1 | 1.0 | 1.0 | 1.0 | 1.0 | 1.1 | 1.0 | 1.0 | 1.0 | 1.0 |
| Pfizer | S6B | 1.0 | 1.0 | 1.0 | 1.0 | 1.0 | 0.9 | 12.5 | 0.9 | 1.0 | 1.0 | 1.0 | 1.0 | 1.0 | 1.1 | 0.9 | 1.0 | 1.0 | 1.0 | 1.0 | 1.0 | 1.0 | 1.0 | 1.0 |
| EuB | | 1.0 | 1.0 | 1.0 | 1.0 | 1.0 | 1.1 | 6.3 | 1.0 | 1.0 | 1.0 | 1.0 | 1.0 | 1.0 | 1.0 | 1.0 | 1.0 | 1.0 | 1.0 | 0.9 | 1.0 | 1.0 | 1.0 | 1.0 |
| Pfizer | S7F | 1.0 | 1.0 | 1.0 | 1.0 | 1.0 | 1.0 | 1.0 | 10.1 | 1.0 | 1.0 | 1.0 | 0.9 | 1.0 | 0.8 | 1.0 | 1.0 | 1.0 | 1.0 | 1.0 | 1.0 | 0.9 | 1.0 | 1.0 |
| EuB | | 1.0 | 1.0 | 1.0 | 1.0 | 1.0 | 1.0 | 1.0 | 8.7 | 1.0 | 1.0 | 1.0 | 1.0 | 1.0 | 1.0 | 1.0 | 1.0 | 1.1 | 1.0 | 1.0 | 1.0 | 1.0 | 1.0 | 1.0 |
| Pfizer | S9V | 0.9 | 0.9 | 0.9 | 1.0 | 1.0 | 0.9 | 0.9 | 0.9 | 0.9 | 0.9 | 17.9 | 1.0 | 1.0 | 1.0 | 0.9 | 0.9 | 1.0 | 0.9 | 0.9 | 1.0 | 1.0 | 0.9 | 1.0 |
| EuB | | 1.0 | 1.0 | 1.0 | 1.0 | 1.0 | 1.0 | 1.0 | 1.0 | 1.0 | 0.9 | 15.5 | 1.0 | 1.0 | 1.1 | 1.0 | 1.0 | 1.0 | 1.0 | 1.0 | 1.0 | 1.0 | 1.0 | 1.0 |
| Pfizer | S14 | 1.0 | 1.0 | 1.0 | 1.0 | 1.0 | 1.0 | 1.0 | 1.0 | 1.0 | 1.0 | 1.0 | 1.0 | 1.0 | 7.0 | 1.0 | 1.0 | 1.0 | 1.0 | 0.9 | 1.0 | 1.0 | 0.9 | 0.9 |
| EuB | | 1.1 | 1.0 | 0.9 | 1.0 | 1.0 | 1.0 | 1.0 | 1.0 | 1.0 | 0.9 | 1.0 | 1.0 | 0.9 | 5.7 | 1.0 | 1.0 | 1.0 | 0.9 | 0.9 | 1.0 | 1.0 | 1.0 | 0.9 |
| Pfizer | S18C | 1.0 | 1.0 | 1.0 | 1.0 | 1.0 | 1.0 | 1.0 | 1.0 | 1.0 | 0.9 | 1.0 | 1.0 | 1.0 | 0.9 | 1.1 | 1.0 | 13.6 | 1.0 | 1.0 | 1.0 | 0.9 | 1.0 | 1.0 |
| EuB | | 1.0 | 1.0 | 1.0 | 1.0 | 1.1 | 1.0 | 1.0 | 1.1 | 1.0 | 1.0 | 1.0 | 1.0 | 1.0 | 1.1 | 1.0 | 1.1 | 8.3 | 1.0 | 1.0 | 1.0 | 1.0 | 1.1 | 1.0 |
| Pfizer | S19A | 1.0 | 0.9 | 1.0 | 1.0 | 1.0 | 1.0 | 0.9 | 0.9 | 1.0 | 1.0 | 1.0 | 0.9 | 0.9 | 1.0 | 0.9 | 0.9 | 0.9 | 12.9 | 0.9 | 1.0 | 1.0 | 13.4 | 1.0 |
| EuB | | 0.9 | 1.0 | 0.9 | 1.0 | 1.0 | 1.0 | 0.9 | 1.0 | 1.0 | 0.9 | 0.9 | 0.9 | 1.0 | 1.0 | 1.0 | 1.0 | 0.9 | 7.0 | 1.0 | 0.9 | 0.9 | 1.0 | 0.9 |
| Pfizer | S19F | 1.0 | 1.0 | 1.0 | 1.0 | 1.0 | 1.0 | 1.0 | 1.0 | 1.0 | 1.0 | 1.0 | 1.0 | 1.0 | 1.0 | 1.0 | 1.0 | 1.0 | 1.0 | 9.9 | 0.9 | 1.0 | 0.9 | 1.0 |
| EuB | | 1.0 | 1.0 | 0.9 | 0.9 | 1.0 | 0.9 | 1.0 | 1.0 | 0.9 | 1.0 | 0.9 | 1.1 | 1.0 | 1.0 | 0.9 | 1.0 | 1.0 | 0.9 | 5.5 | 1.0 | 1.0 | 1.0 | 0.9 |
| EuB | S22F | 1.0 | 1.0 | 1.0 | 0.9 | 0.9 | 0.9 | 0.9 | 0.9 | 0.9 | 0.9 | 0.9 | 1.0 | 0.9 | 0.8 | 0.9 | 0.9 | 0.9 | 0.9 | 0.9 | 0.9 | 6.0 | 0.9 | 0.9 |
| Pfizer | S23F | 1.0 | 1.0 | 1.0 | 1.0 | 1.0 | 1.0 | 1.0 | 1.0 | 1.0 | 1.0 | 1.0 | 0.9 | 1.0 | 0.9 | 1.0 | 1.0 | 1.0 | 1.0 | 0.9 | 1.0 | 1.0 | 16.0 | 1.1 |
| EuB | | 0.9 | 0.9 | 0.9 | 0.9 | 0.9 | 0.9 | 1.0 | 0.9 | 0.9 | 0.9 | 0.9 | 0.9 | 0.9 | 0.9 | 0.9 | 0.9 | 0.9 | 0.9 | 0.9 | 0.9 | 0.9 | 8.8 | 0.9 |

## Slide 3
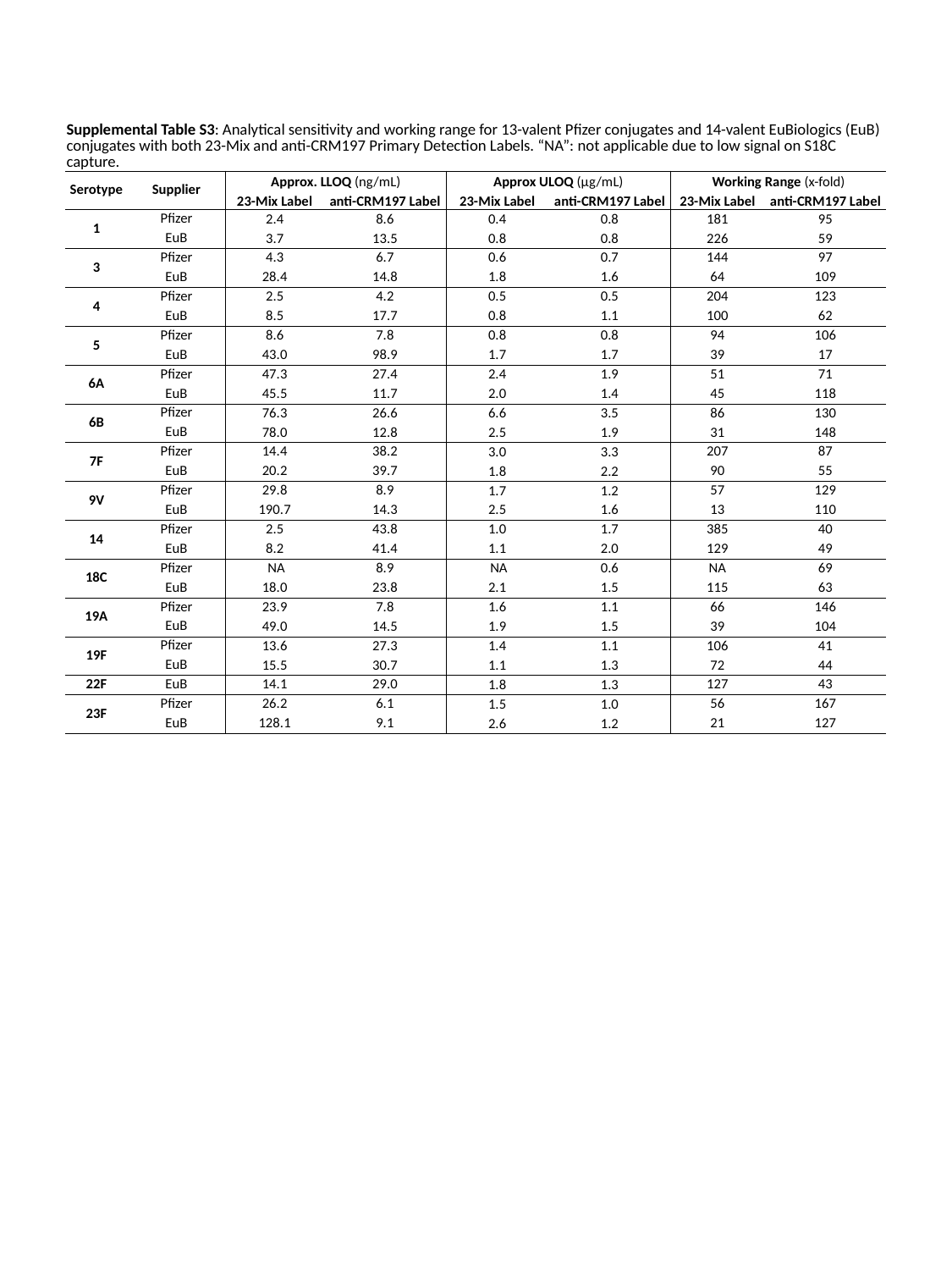

| Supplemental Table S3: Analytical sensitivity and working range for 13-valent Pfizer conjugates and 14-valent EuBiologics (EuB) conjugates with both 23-Mix and anti-CRM197 Primary Detection Labels. “NA”: not applicable due to low signal on S18C capture. | | | | | | | |
| --- | --- | --- | --- | --- | --- | --- | --- |
| Serotype | Supplier | Approx. LLOQ (ng/mL) | | Approx ULOQ (μg/mL) | | Working Range (x-fold) | |
| | | 23-Mix Label | anti-CRM197 Label | 23-Mix Label | anti-CRM197 Label | 23-Mix Label | anti-CRM197 Label |
| 1 | Pfizer | 2.4 | 8.6 | 0.4 | 0.8 | 181 | 95 |
| | EuB | 3.7 | 13.5 | 0.8 | 0.8 | 226 | 59 |
| 3 | Pfizer | 4.3 | 6.7 | 0.6 | 0.7 | 144 | 97 |
| | EuB | 28.4 | 14.8 | 1.8 | 1.6 | 64 | 109 |
| 4 | Pfizer | 2.5 | 4.2 | 0.5 | 0.5 | 204 | 123 |
| | EuB | 8.5 | 17.7 | 0.8 | 1.1 | 100 | 62 |
| 5 | Pfizer | 8.6 | 7.8 | 0.8 | 0.8 | 94 | 106 |
| | EuB | 43.0 | 98.9 | 1.7 | 1.7 | 39 | 17 |
| 6A | Pfizer | 47.3 | 27.4 | 2.4 | 1.9 | 51 | 71 |
| | EuB | 45.5 | 11.7 | 2.0 | 1.4 | 45 | 118 |
| 6B | Pfizer | 76.3 | 26.6 | 6.6 | 3.5 | 86 | 130 |
| | EuB | 78.0 | 12.8 | 2.5 | 1.9 | 31 | 148 |
| 7F | Pfizer | 14.4 | 38.2 | 3.0 | 3.3 | 207 | 87 |
| | EuB | 20.2 | 39.7 | 1.8 | 2.2 | 90 | 55 |
| 9V | Pfizer | 29.8 | 8.9 | 1.7 | 1.2 | 57 | 129 |
| | EuB | 190.7 | 14.3 | 2.5 | 1.6 | 13 | 110 |
| 14 | Pfizer | 2.5 | 43.8 | 1.0 | 1.7 | 385 | 40 |
| | EuB | 8.2 | 41.4 | 1.1 | 2.0 | 129 | 49 |
| 18C | Pfizer | NA | 8.9 | NA | 0.6 | NA | 69 |
| | EuB | 18.0 | 23.8 | 2.1 | 1.5 | 115 | 63 |
| 19A | Pfizer | 23.9 | 7.8 | 1.6 | 1.1 | 66 | 146 |
| | EuB | 49.0 | 14.5 | 1.9 | 1.5 | 39 | 104 |
| 19F | Pfizer | 13.6 | 27.3 | 1.4 | 1.1 | 106 | 41 |
| | EuB | 15.5 | 30.7 | 1.1 | 1.3 | 72 | 44 |
| 22F | EuB | 14.1 | 29.0 | 1.8 | 1.3 | 127 | 43 |
| 23F | Pfizer | 26.2 | 6.1 | 1.5 | 1.0 | 56 | 167 |
| | EuB | 128.1 | 9.1 | 2.6 | 1.2 | 21 | 127 |

## Slide 4
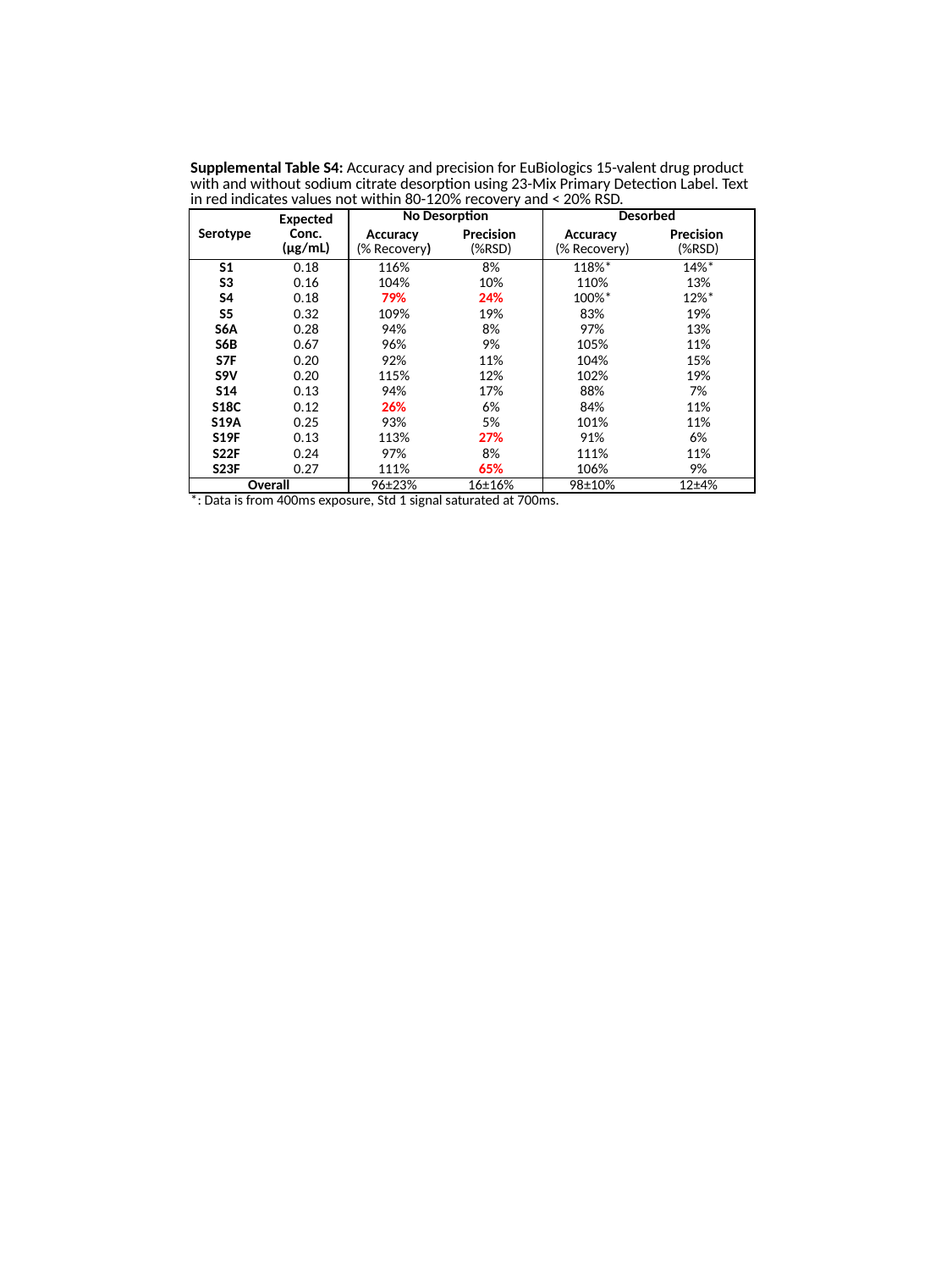

| Supplemental Table S4: Accuracy and precision for EuBiologics 15-valent drug product with and without sodium citrate desorption using 23-Mix Primary Detection Label. Text in red indicates values not within 80-120% recovery and < 20% RSD. | | | | | |
| --- | --- | --- | --- | --- | --- |
| Serotype | Expected Conc. (μg/mL) | No Desorption | | Desorbed | |
| | | Accuracy (% Recovery) | Precision (%RSD) | Accuracy (% Recovery) | Precision (%RSD) |
| S1 | 0.18 | 116% | 8% | 118%\* | 14%\* |
| S3 | 0.16 | 104% | 10% | 110% | 13% |
| S4 | 0.18 | 79% | 24% | 100%\* | 12%\* |
| S5 | 0.32 | 109% | 19% | 83% | 19% |
| S6A | 0.28 | 94% | 8% | 97% | 13% |
| S6B | 0.67 | 96% | 9% | 105% | 11% |
| S7F | 0.20 | 92% | 11% | 104% | 15% |
| S9V | 0.20 | 115% | 12% | 102% | 19% |
| S14 | 0.13 | 94% | 17% | 88% | 7% |
| S18C | 0.12 | 26% | 6% | 84% | 11% |
| S19A | 0.25 | 93% | 5% | 101% | 11% |
| S19F | 0.13 | 113% | 27% | 91% | 6% |
| S22F | 0.24 | 97% | 8% | 111% | 11% |
| S23F | 0.27 | 111% | 65% | 106% | 9% |
| Overall | | 96±23% | 16±16% | 98±10% | 12±4% |
| \*: Data is from 400ms exposure, Std 1 signal saturated at 700ms. | | | | | |

## Slide 5
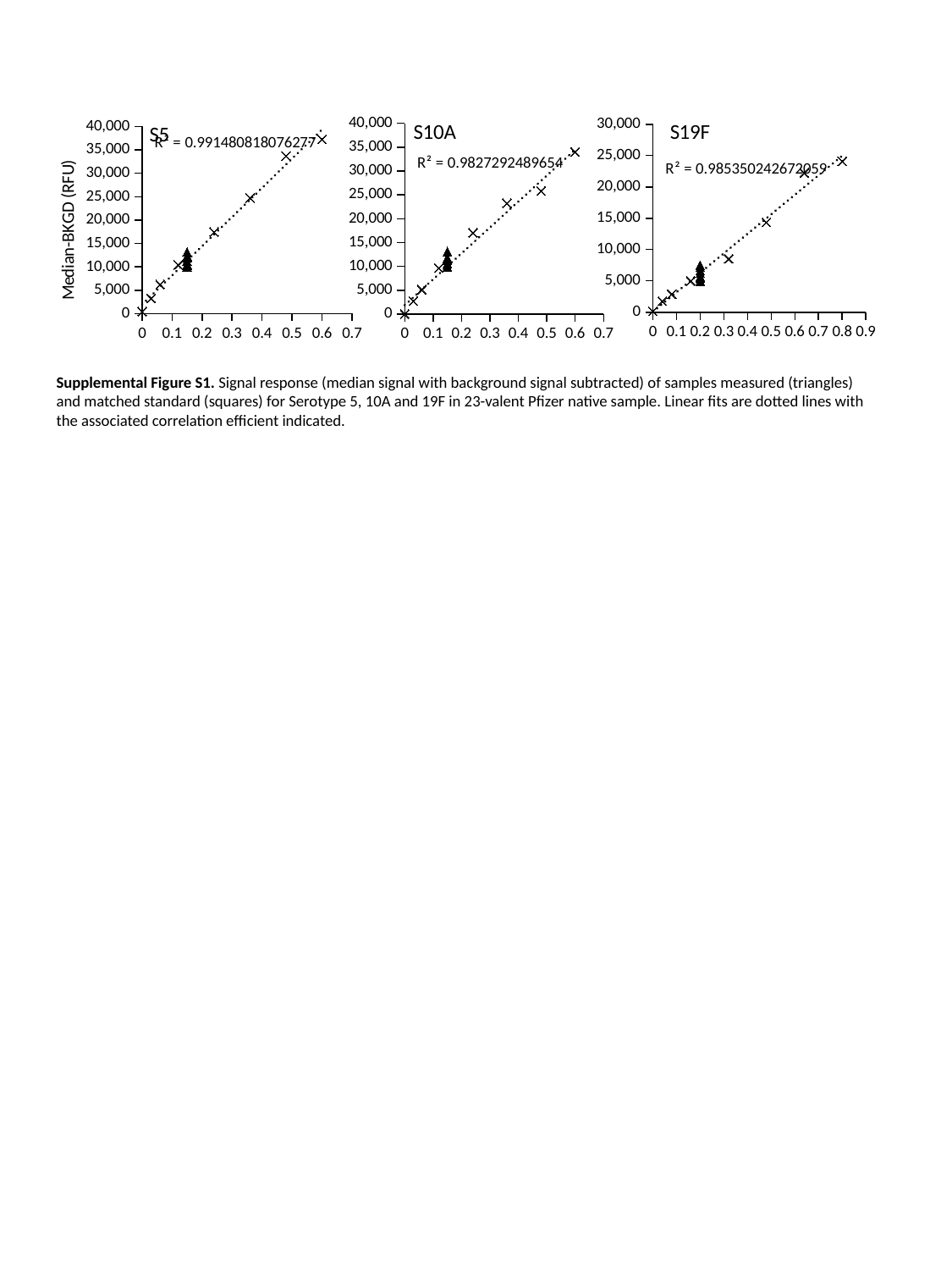

### Chart: S10A
| Category | Standard | Reps |
|---|---|---|
### Chart: S19F
| Category | Standard | Reps |
|---|---|---|
### Chart: S5
| Category | Standard | Reps |
|---|---|---|Supplemental Figure S1. Signal response (median signal with background signal subtracted) of samples measured (triangles) and matched standard (squares) for Serotype 5, 10A and 19F in 23-valent Pfizer native sample. Linear fits are dotted lines with the associated correlation efficient indicated.

## Slide 6
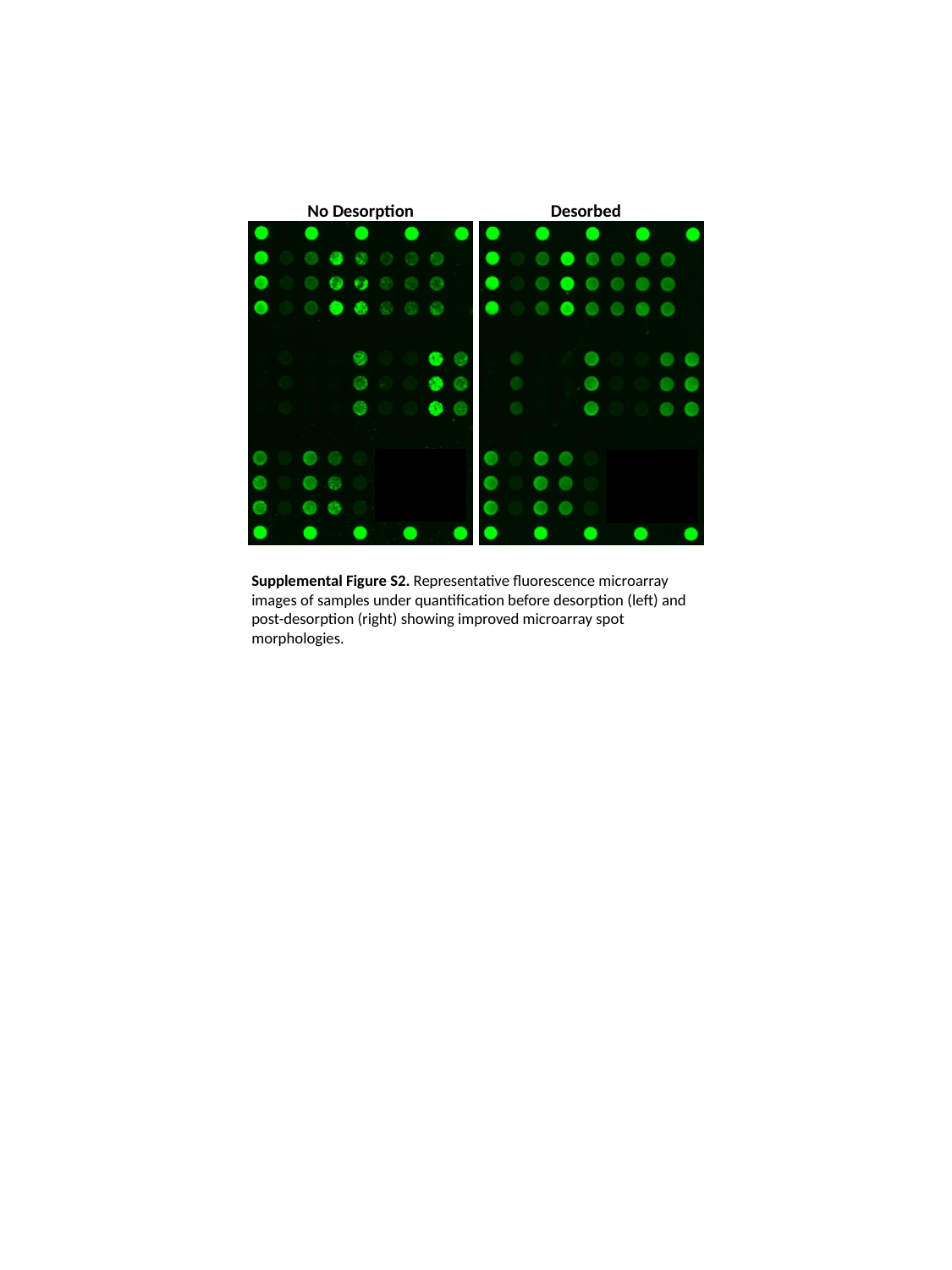

No Desorption
Desorbed
Supplemental Figure S2. Representative fluorescence microarray images of samples under quantification before desorption (left) and post-desorption (right) showing improved microarray spot morphologies.
