# Supplementary material for: 30-Minute Highly Multiplexed VaxArray Immunoassay for Pneumococcal Vaccine Antigen Characterization
Source: Vaccines (Basel). 2022 Nov 19;10(11):1964. doi: 10.3390/vaccines10111964 (PMC9693921; doi:10.3390/vaccines10111964)
Supplement: Supplementary file 1 [file vaccines-10-01964-s001.zip › Legends of Supplemental Figures and Tables.pdf]

Legends of Figures and Tables

Supplemental Figure S1. Signal response (median signal with background signal subtracted) of samples measured (triangles) and matched standard (squares) for Serotype 5, 10A and 19F in 23-valent Pfizer native sample. Linear fits are dotted lines with the associated correlation efficient indicated.

Supplemental Figure S2. Representative fluorescence microarray images of samples under quantification before desorption (left) and post-desorption (right) showing improved microarray spot morphologies.

Supplemental Table S1: Reactivity of conjugates using 23-Mix Primary Detection Label. Signal to blank ratios (S/Bl) generated on the VaxArray 23-valent Pneumococcal Assay for monovalent Pfizer- and EuBiologics (EuB)-provided conjugates at 2 μg/mL using the 23-Mix Primary Detection Label. Columns represent each serotype-specific capture antibody, and rows represent each conjugate tested. Text in bold green shows S/Bl>3 generated for the matched conjugate and capture antibody. Text in bold red shows S/Bl<3 generated for the matched conjugate and capture antibody indicating low reactivity. Text in bold black shows S/Bl>3 generated on off-target capture antibody indicating cross-reactivity.

Supplemental Table S2: Reactivity of conjugates using anti-CRM197 Primary Detection Label. Signal to blank ratios (S/Bl) generated on the VaxArray 23-valent Pneumococcal Assay for monovalent Pfizer- and EuBiologics (EuB)-provided conjugates at 2 μg/mL labeled with anti-CRM197 Primary Detection Label. Columns represent each serotype-specific capture antibody, and rows represent each conjugate tested. Text in bold green shows S/Bl>3 generated for the matched conjugate and capture antibody. Text in bold black shows S/Bl>3 generated on off-target capture antibody indicating cross-reactivity.

Supplemental Table S3: Analytical sensitivity and working range for 13-valent Pfizer conjugates and 14-valent EuBiologics (EuB) conjugates with both 23-Mix and anti-CRM197 Primary Detection Labels. “NA”: not applicable due to low signal on S18C capture.

Supplemental Table S4: Accuracy and precision for EuBiologics 15-valent drug product with and without sodium citrate desorption using 23-Mix Primary Detection Label. Text in red indicates values not within 80-120% recovery and < 20% RSD.
