# Supplementary figures and images for: 30-Minute Highly Multiplexed VaxArray Immunoassay for Pneumococcal Vaccine Antigen Characterization
Source: Vaccines (Basel). 2022 Nov 19;10(11):1964. doi: 10.3390/vaccines10111964 (PMC9693921; doi:10.3390/vaccines10111964)

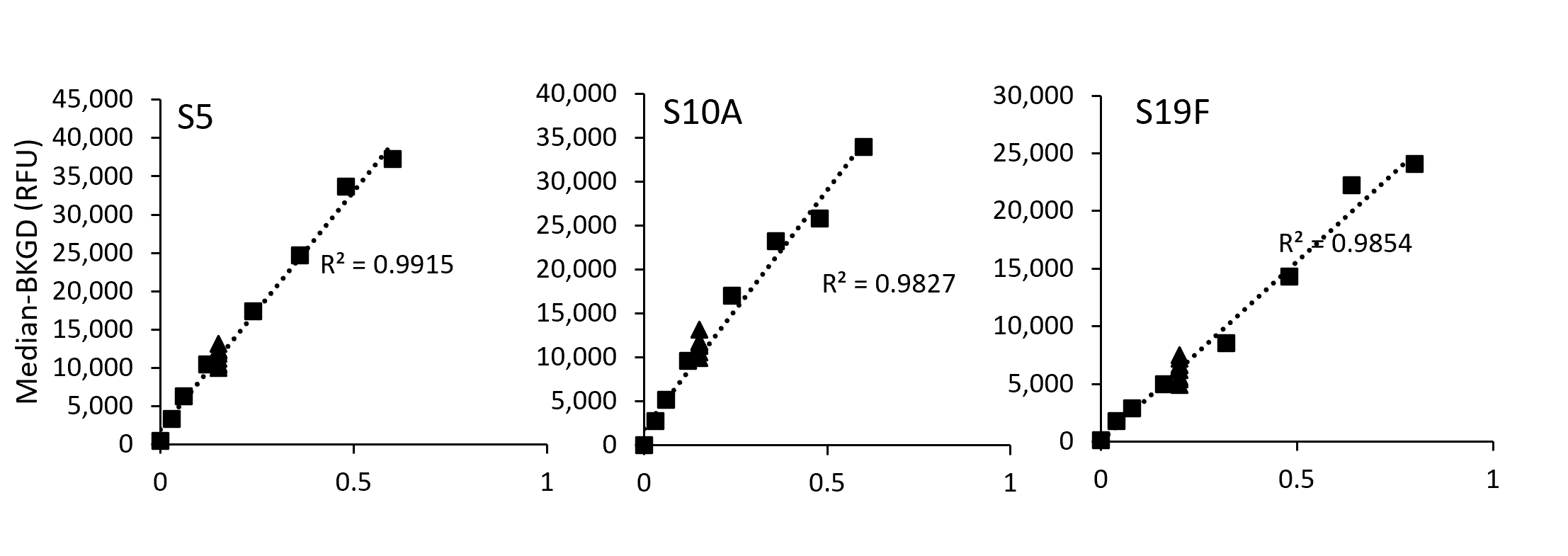

Supplement: Supplementary file 1 [file vaccines-10-01964-s001.zip › Supplemental Figure S1.png]

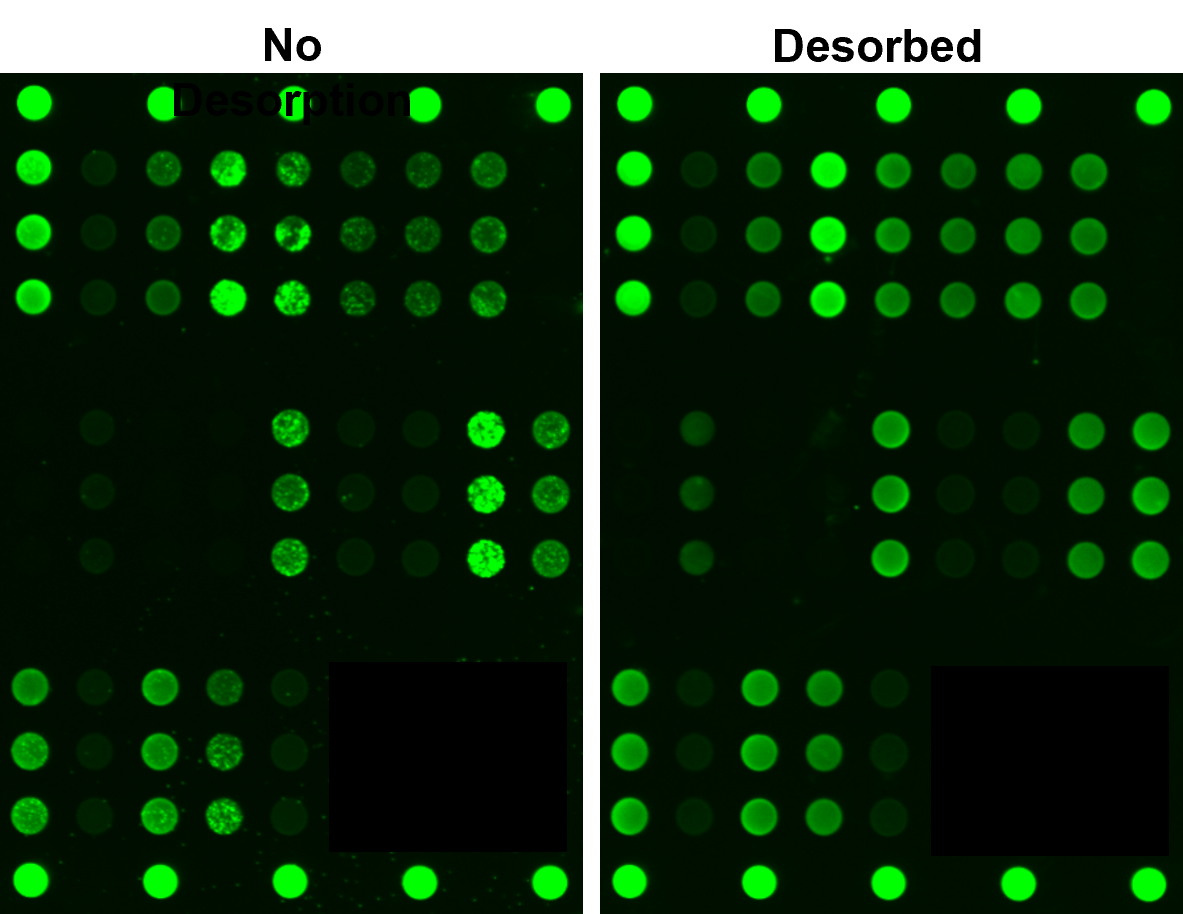

Supplement: Supplementary file 1 [file vaccines-10-01964-s001.zip › Supplemental Figure S2.png]

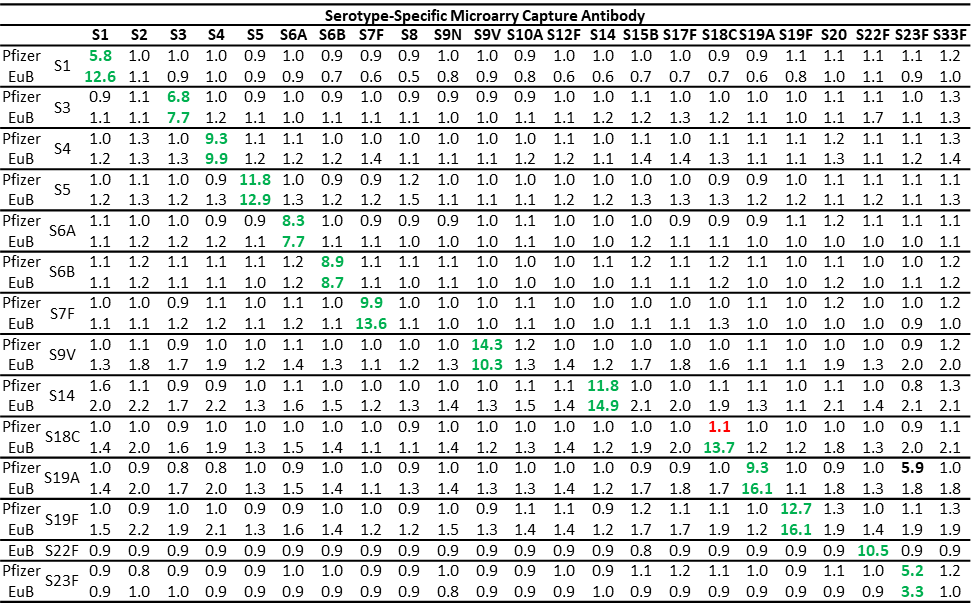

Supplement: Supplementary file 1 [file vaccines-10-01964-s001.zip › Supplemental Table S1.png]

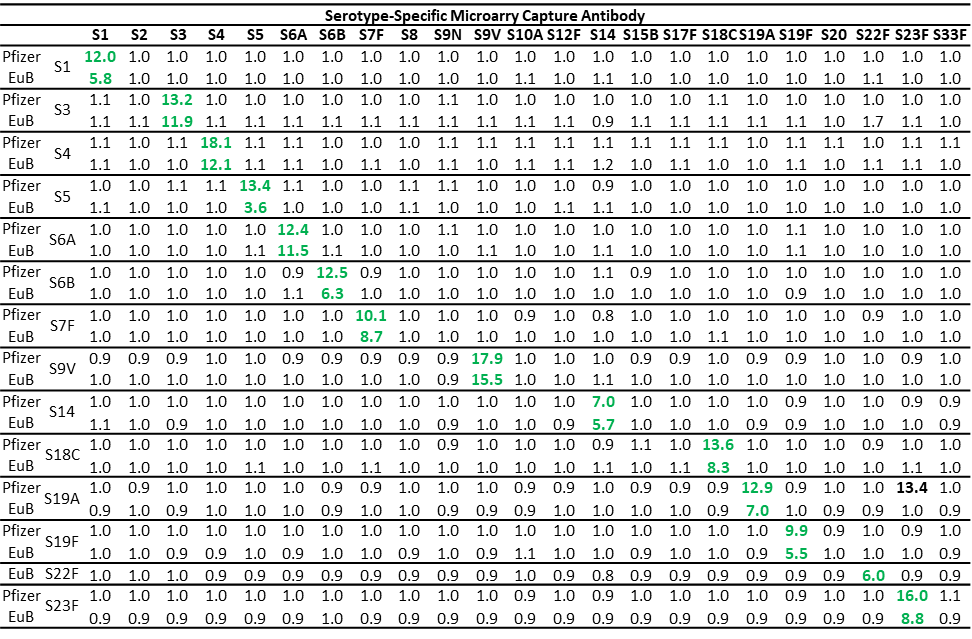

Supplement: Supplementary file 1 [file vaccines-10-01964-s001.zip › Supplemental Table S2.png]

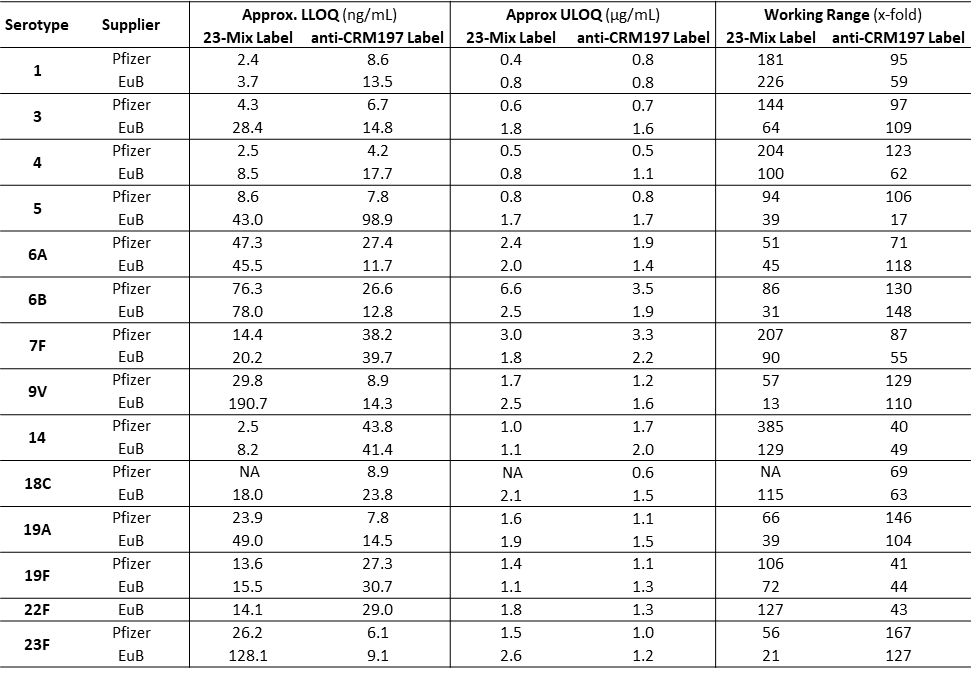

Supplement: Supplementary file 1 [file vaccines-10-01964-s001.zip › Supplemental Table S3.png]

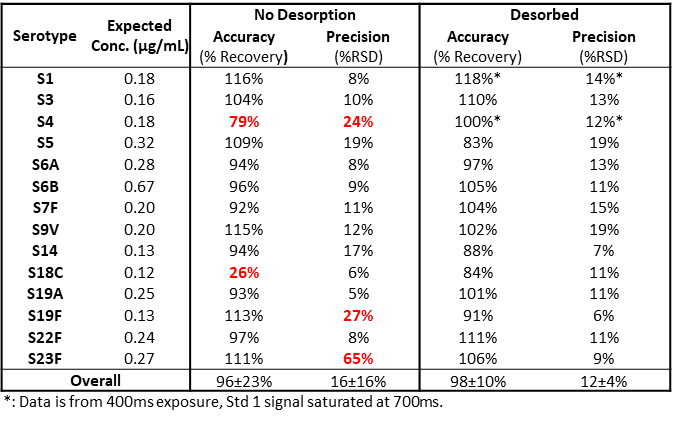

Supplement: Supplementary file 1 [file vaccines-10-01964-s001.zip › Supplemental Table S4.png]
